# Supplementary material for: Recurrent photic zone euxinia limited ocean oxygenation and animal evolution during the Ediacaran
Source: Nat Commun. 2023 Jul 3;14:3920. doi: 10.1038/s41467-023-39427-z (PMC10318064; doi:10.1038/s41467-023-39427-z)
Supplement: Supplementary file 1 — Supplementary Information [file 41467_2023_39427_MOESM1_ESM.pdf]

Supplementary Information

Recurrent photic zone euxinia limited ocean oxygenation and animal evolution during the Ediacaran

Wang Zheng[1], Anwen Zhou[1,2], Swapan K. Sahoo*[3], Morrison R. Nolan[4], Chadlin M. Ostrander[5,6], Ruoyu Sun[1], Ariel D. Anbar[7], Shuhai Xiao[4], Jiubin Chen*[1]

[1]*School of Earth System Science, Institute of Surface-Earth System Science, Tianjin University, 300072 Tianjin, China*
[2]*Department of Earth, Ocean and Atmospheric Science and National High Magnetic Field Laboratory, Florida State University, Tallahassee, FL 32306, USA*
[3]*Equinor US, Houston, TX, USA*
[4]*Department of Geosciences, Virginia Tech, Blacksburg, VA, USA*
[5]*Department of Marine Chemistry and Geochemistry, Woods Hole Oceanographic Institution, Woods Hole, MA 02543, USA*
[6]*Department of Geology and Geophysics, University of Utah, Salt Lake City, UT, 84112, USA*
[7]*School of Earth and Space Exploration, Arizona State University, Tempe, AZ 85287, USA*

*Corresponding author:
S. K. Sahoo: swas@equinor.com
J. Chen: jbchen@tju.edu.cn

Contains Supplementary Text S1-S6, Figures S1-S6, Tables S1-S3

Text S1. Correlations of Hg concentration with proxies of major host phases

The correlations between THg and typical host phases of Hg in sediments (i.e., organic matter, sulfide and clay minerals) are often examined to help determine the mechanism of Hg enrichment in sediments[1]. Since the host phases could be different under different redox conditions[1], it is reasonable to examine the correlations between THg and host phases for OOE and non-OOE intervals separately (Figure S1). In WH section, THg shows strong correlations with both total organic carbon (TOC) and pyrite S (S_{py}) in both Member II and IV OOE intervals, but the correlations with S_{py} ($R^2 = 0.77$ and 0.69, respectively) are even stronger than those with TOC ($R^2 = 0.47$ and 0.62, respectively). However, the correlations with both TOC and S_{py} are very weak in the Member III OOE interval, partly due to the scarcity of data points in this interval. In the non-OOE intervals of WH section, THg still shows a significant correlation with TOC ($R^2 = 0.47$, $P < 0.001$), but only a weak correlation with S_{py} ($R^2 = 0.23$, $P = 0.002$). The correlations with Al (a typical proxy of clay mineral) are insignificant in the entire WH section. For TY and YJ sections, the correlations with TOC and S_{py} are mostly insignificant except a relatively good correlation with TOC in the Member II OOE interval ($R^2 = 0.36$, $P < 0.001$) of TY section. Interestingly, there are significant correlations with Al in the Member IV OOE interval of TY ($R^2 = 0.87$, $P < 0.01$) and Member II OOE interval of YJ section ($R^2 = 0.45$, $P = 0.02$). In WA section, THg shows strong correlations with both TOC ($R^2 = 0.85$, $P < 0.001$) and Al ($R^2 = 0.83$, $P < 0.001$), but no correlation with S_{py} (Figure S1).

Text S2. Evaluation of potential diagenetic and metamorphic alteration

The Hg isotope compositions in the study sections are unlikely altered by post-depositional processes based on the following evidence. First, recent experimental studies showed that Hg MDF and MIF values in black shales are generally well preserved below a temperature of ~250 °C, and that higher pressures actually favor the retention of Hg in sediments[2,3]. Above 250 °C, substantial Hg loss may occur, and δ^{202}Hg may show detectable increases (>0.1‰) due to the preferential loss of lighter isotopes, but MIF would remain unchanged even at temperatures as high as 800 °C[2,3]. Therefore, the Hg MIF in Doushantuo shales should represent the indigenous MIF signals of seawater. Second, significant alteration of δ^{202}Hg is also unlikely considering its strong correlation with all MIF values in all study sections (Figure 3a, S2 and S3). These correlations cannot be produced by diagenetic or metamorphic alteration under high temperature or pressure[2,3]. Moreover, Deng et al. compared Hg MDF in rocks experienced different grades of metamorphism and found no systematic MDF changes during metamorphism[4]. Third, the cyclic pattern of Hg isotopes in WH is generally in sync with other redox proxies for the same section (i.e., RSE, Fe speciation, $\delta^{34}S_{pyrite}$, δ^{98}Mo, δ^{53}Cr, and ε^{205}Tl)[5–8], and previous studies concluded that the cyclic pattern of these proxies was unlikely caused by diagenetic alteration[6–8].

Text S3. A compilation of Hg isotope data of modern samples

Below is a summary of background Hg isotope signatures of modern samples, which may serve as analogs of major Hg sources to ancient sedimentary rocks (Figure S5). The "background" means these Hg sources are not directly contaminated by nearby point-source emissions, but represent the global background value. The terrestrial Hg in modern environment

is primarily associated with terrestrial organic matter (OM) such as soil and biomass[9], which have significantly negative Δ^{199}Hg (−0.25 ± 0.12‰, 1SD), near zero Δ^{200}Hg (0.00 ± 0.04‰, 1SD) and negative δ^{202}Hg (−1.81 ± 0.68‰, 1SD)[10–19]. Atmospheric deposition to ocean includes the depositions of gaseous Hg(0) and oxidized Hg(II) species (primarily in the form of precipitation and particulate Hg). Atmospheric gaseous Hg(0) shows less negative Δ^{199}Hg (−0.20 ± 0.09‰, 1SD) than terrestrial Hg, more negative Δ^{200}Hg (−0.05 ± 0.04‰, 1SD) and predominantly positive δ^{202}Hg (0.43 ± 0.51‰, 1SD)[11,13,17,20–25], whereas atmospheric oxidized Hg(II) species show positive Δ^{199}Hg (0.41 ± 0.32‰, 1SD), positive Δ^{200}Hg (0.12 ± 0.11‰, 1SD) and negative δ^{202}Hg (−0.84 ± 0.55‰, 1SD)[11,13,17,20,24,26–29]. The Hg isotope compositions of modern open ocean seawater (including Mediterranean Sea, Atlantic Ocean and Pacific Ocean) are proposed to reflect the mixing between atmospheric Hg(0) and Hg(II) depositions, and thus show intermediate Δ^{199}Hg (0.09 ± 0.04‰, 1SD), Δ^{200}Hg (0.03 ± 0.04‰, 1SD) and δ^{202}Hg (−0.33 ± 0.41‰, 1SD) values between atmospheric Hg(0) and Hg(II)[30]. Modern open ocean sediments (mostly from Mediterranean Sea) show similar Δ^{199}Hg (0.09 ± 0.03‰, 1SD) and Δ^{200}Hg (0.03 ± 0.02, 1SD) as the seawater, but more negative δ^{202}Hg (−1.21 ± 0.58‰, 1SD) than seawater[30–33]. Geogenic Hg is represented by direct measurements of volcanic Hg emission, which has near-zero Δ^{199}Hg (0.03 ± 0.05‰, 1SD), near-zero Δ^{200}Hg (0.02 ± 0.05, 1SD), and negative δ^{202}Hg (−0.62 ± 0.69‰, 1SD)[34–36].

Text S4. The simplified Hg isotope box-model

To quantitatively estimate the effect of terrestrial weathering and local volcanism on Hg budget and Δ^{199}Hg in the ocean, we constructed a simplified Hg isotope box-model for the studied time interval (Figure S6) based on the previously published Hg isotope box-models[37,38]. The algorithm is based on ordinary differential equations according to Hg mass balance and Hg isotope mass balance constraints[37], which are applied for three coupled reservoirs: atmospheric Hg(0), atmospheric Hg(II) and ocean Hg. The mass transfer of Hg masses and Hg isotope signatures between boxes (reservoirs) are controlled by first order rate coefficients (k, yr^{-1}), which are mostly derived from the reservoir sizes and fluxes of the well-known modern Hg cycle[39–41].

The initial reservoir sizes of the model are taken from Amos et al., 2013[42] where atmospheric Hg is speciated as Hg(0) (80%) and Hg(II) (20%). The fluxes of atmospheric Hg(0) and Hg(II) deposition are prescribed as 50%:50% to reflect the recent advance[30]. Before running the model, the ocean reservoir is prescribed to be in a pseudo-steady-state in both Hg fluxes and isotope compositions (i.e., input=output). The background subaerial volcanic Hg emission flux and background submarine hydrothermal flux are assumed to be 150 Mg a^{-1}, and 100 Mg a^{-1}, respectively, according to recent UNEP Hg assessment report[43]. The local subaerial volcanic Hg emission flux is assumed to be 10 Mg a^{-1}. The Δ^{199}Hg of volcanic emissions and hydrothermal discharges are both assumed to be 0.00‰[35,44]. We use a lateral water advection flux (representing other water bodies in exchange with our modeled ocean) to balance the Hg fluxes and isotope compositions of the modeled ocean. Given the consistent background atmospheric Hg(0) and Hg(II) isotope composition observed in modern days[30], which are around 0.40‰ and −0.20‰ in Δ^{199}Hg, respectively (Text S3), we simplified our system to a simple end-member mixing model for marine Hg isotope compositions, which is similar to the

treatment in a published box-model that was applied to simulate terrestrial and volcanic Hg inputs during end-Permian mass extinction[45].

Starting from the reservoir Hg budgets and Hg isotope values at the pseudo-steady-state, we model how the marine Hg budgets and Δ^{199}Hg values respond to increases of the intensities of terrestrial weathering and local volcanism (which was likely a major source of Hg in E3, see Text S5) during the three E intervals. Our modeling suggests an increase of terrestrial weathering rate by 10-50 times can only account for <0.03‰ decrease in Δ^{199}Hg (Figure 4), which is insufficient to explain the ~0.2‰ negative shifts observed in E1, E2 and E3. Similarly, an increase of local volcanic Hg by 100 times in E3 also only resulted in <0.03‰ decease of Δ^{199}Hg. The reason for the insensitivity of marine Δ^{199}Hg to the variations of terrestrial weathering and local volcanism is because atmospheric Hg pool would be simultaneously increased in response to enhanced Hg re-emission from the marine Hg pool. The re-emitted Hg would undergo atmospheric redox transformations that produce a net positive Δ^{199}Hg signal for atmospheric Hg species, which then deposit back to the surface ocean. Thus the enhanced re-emission and re-deposition of Hg with positive Δ^{199}Hg following enhanced terrestrial weathering or local volcanism eventually counteracted the negative shift of Δ^{199}Hg caused by terrestrial or volcanic inputs. Therefore, our model suggests that terrestrial weathering and local volcanic emissions are insufficient to cause the large, negative excursion of Δ^{199}Hg during E intervals.

Text S5. Evidence of volcanism and upwelling of Hg associated with organic matter in Member IV of WH and TY sections

The E3 interval (Member IV OOE) of both WH and TY sections show distinct patterns of Hg enrichment and Hg isotopes compared to E1 and E2 intervals (Member II and III OOEs). Unlike the other members, the Hg isotope compositions of Member IV in both WH and TY sections do not exactly follow the negative correlation between Δ^{199}Hg and δ^{202}Hg (Figure S2), suggesting that Hg in Member IV has different sources or was affected by different processes compared to other members. In WH section, the E3 interval shows near-zero Δ^{199}Hg with a variable but mostly negative δ^{202}Hg. These isotope signatures are consistent with local volcanic Hg input[46]. It is also the only interval that shows a strong Hg enrichment marked by simultaneous increases of raw Hg concentration and all normalized ratios (Hg/TOC, Hg/S_{py}, and Hg/Al) (see the "Result" section), suggesting a strong input of external Hg that was decoupled from major host phases. Similar high Hg enrichment was also reported for Member IV of the coeval Jiulongwan section from Yangtze Gorges area, South China[47,48], which was located in a restricted shelf lagoon setting[49], suggesting that the Hg enrichment was widespread during this period. Such anomalous Hg enrichment has been frequently observed in Phanerozoic sedimentary rocks deposited during mass extinction events with coeval large igneous provinces (LIPs) or arc volcanism[50–53], because volcanic emission is the largest natural source of Hg[54]. Thus it is plausible that Member IV records Hg input from large-scale volcanic emission. This is supported by the abundant volcanic ash beds widely distributed in the uppermost Doushantuo Formation in Yangtze Gorges area[55].

In contrast, the E3 interval of TY section shows positive Δ^{199}Hg (up to ~0.2‰) and almost

invariable negative $\delta^{202}Hg$ (~ −1.0‰) (Figure S2). These isotope signatures are different from local volcanic input (MIF=0), but are consistent with those of Hg associated with dissolved organic matter (Hg-OM) upwelling from deep ocean[48,56]. The isotope signatures of Hg-OM are hypothesized to resemble open ocean sediments, which have similar Hg isotope signatures as the E3 interval of TY section[31]. Thus the high Hg content in Member IV of TY section may be driven by upwelling of Hg-OM rather than volcanic input. The different Hg sources between Member IV in TY and WH sections are likely due to their different locations. TY is a shallower section, where the upwelling OM is more likely to meet the chemocline and be oxidized by dissolved oxygen, and thus Hg-OM is more likely to be released to sediments at TY section. Similar Hg isotope signatures have been also reported for Doushantuo Member IV in another upper slope section[57] and shallow shelf lagoon sections in South China[48,56].

Alternative sources of the high Hg in Member IV are submarine hydrothermal emission. However, Hg from submarine hydrothermal emission tend to settle quickly in the vicinity of the emission source and thus a declining concentration gradient is expected from the basin to shelf. This is not observed in the Doushantuo shale, as the upper slope TY section has much higher THg than the lower slope WH section. Furthermore, hydrothermal Hg has predominantly near-zero MIF[58], but the Hg MIF values in WH, TY and Jiulongwan show both zero and positive values (up to ~0.2‰ in TY).

Text S6. Sample preparation for Hg isotope analysis

All sample processing and Hg isotope analyses were conducted at Tianjin University. Briefly, powdered samples were weighed into 30 ml Teflon beakers and digested with a mixture of trace metal grade concentrated acids containing HNO_3, HCl, and BrCl with a volume ratio of 12:6:1 at ~100 °C for 48 hours. Then the digested samples were centrifuged to remove solid residues. Afterwards, an anion-exchange chromatographic method was applied to separate Hg from matrix[59]. Briefly, the centrifuged solutions were loaded onto columns containing anion exchange resin AG1-X4 (200-400 mesh, Bio Rad). After rinsing with 2M HCl to remove the matrix, Hg was eluted with 12ml 0.5M HNO_3 + 0.05% L-cysteine, and then digested with 0.2 M BrCl prior to isotope analysis. The Hg concentrations in the eluted solutions were determined by cold vapor atomic fluorescence spectrometry (CV-AFS, Tekran 2600, Tekran Instruments Corporation). The overall Hg yield of the digestion and anion-exchange chromatographic procedures was calculated by comparing the amount of Hg in the eluted solution with those measured by Lumex, and the yield for all samples was 102 ± 9% (2SD, n = 95).

Figure S1. Correlations of Hg concentration with major host phases, i.e., organic matter (represented by TOC) (Panel A, D, G, J and M), sulfide mineral (represented by pyrite S) (Panel B, E, H, K and N), and clay mineral (represented by Al) (Panel C, F, I, L and O) for WH, TY, YJ, and WA sections.

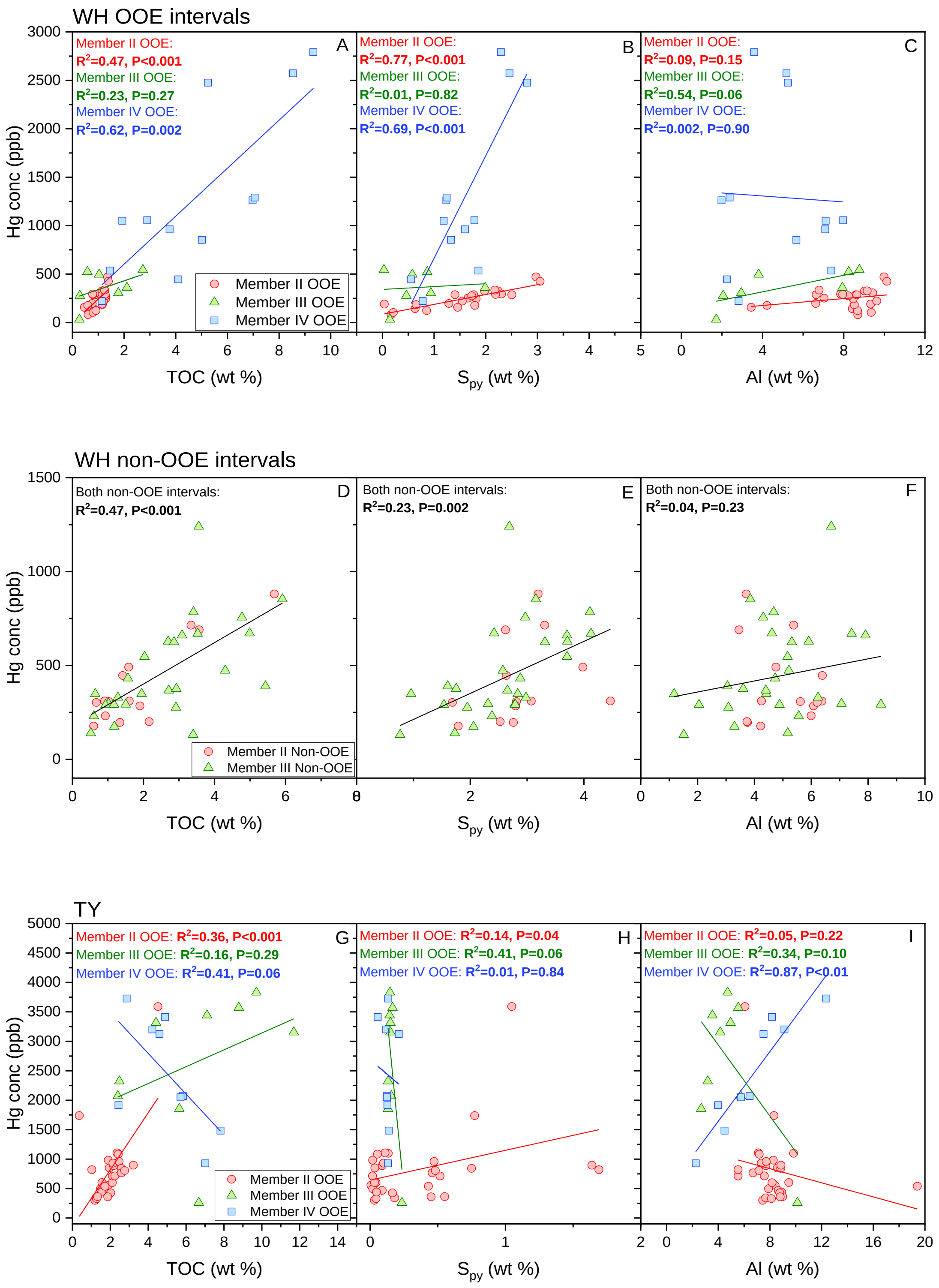

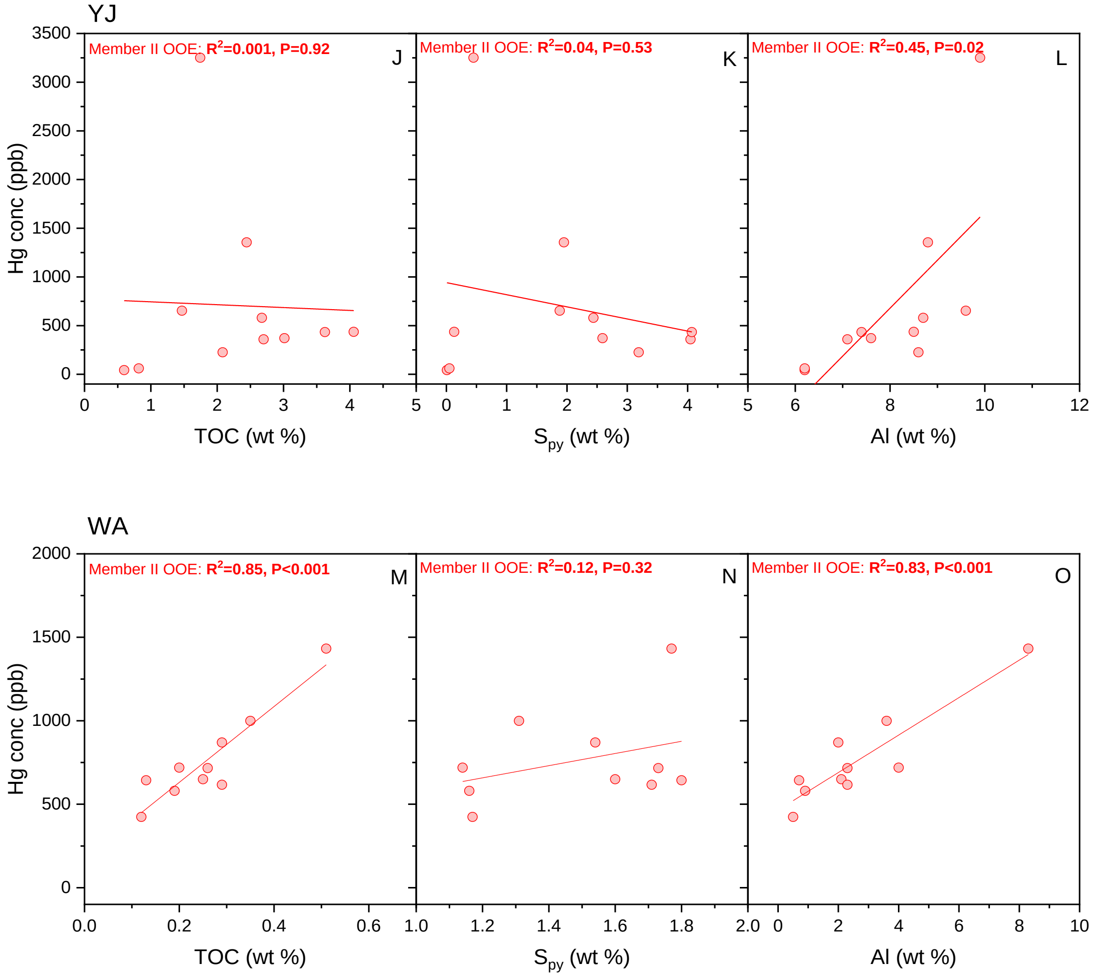

YJ
Member II OOE: R2=0.001, P=0.92
J
Member II OOE: R2=0.04, P=0.53
K
Member II OOE: R2=0.45, P=0.02
L
Hg conc (ppb)
TOC (wt %)
Spy (wt %)
Al (wt %)
WA
Member II OOE: R2=0.85, P<0.001
M
Member II OOE: R2=0.12, P=0.32
N
Member II OOE: R2=0.83, P<0.001
O
Hg conc (ppb)
TOC (wt %)
Spy (wt %)
Al (wt %)

Figure S2. The relationship between Δ^{199}Hg and δ^{202}Hg for a) WA, b) TY, c) WH and d) YJ sections. The black lines are linear regressions for Δ^{199}Hg vs. δ^{202}Hg, and the slopes are: WA (−0.09 ± 0.02, 1SE, R^2 = 0.72, P = 9E-4), TY (−0.09 ± 0.01, 1SE, R^2 = 0.84, P = 1E-10), WH (−0.09 ± 0.02, 1SE, R^2 = 0.47, P = 0) and YJ (−0.09 ± 0.01, 1SE, R^2 = 0.81, P = 6E-5), respectively.

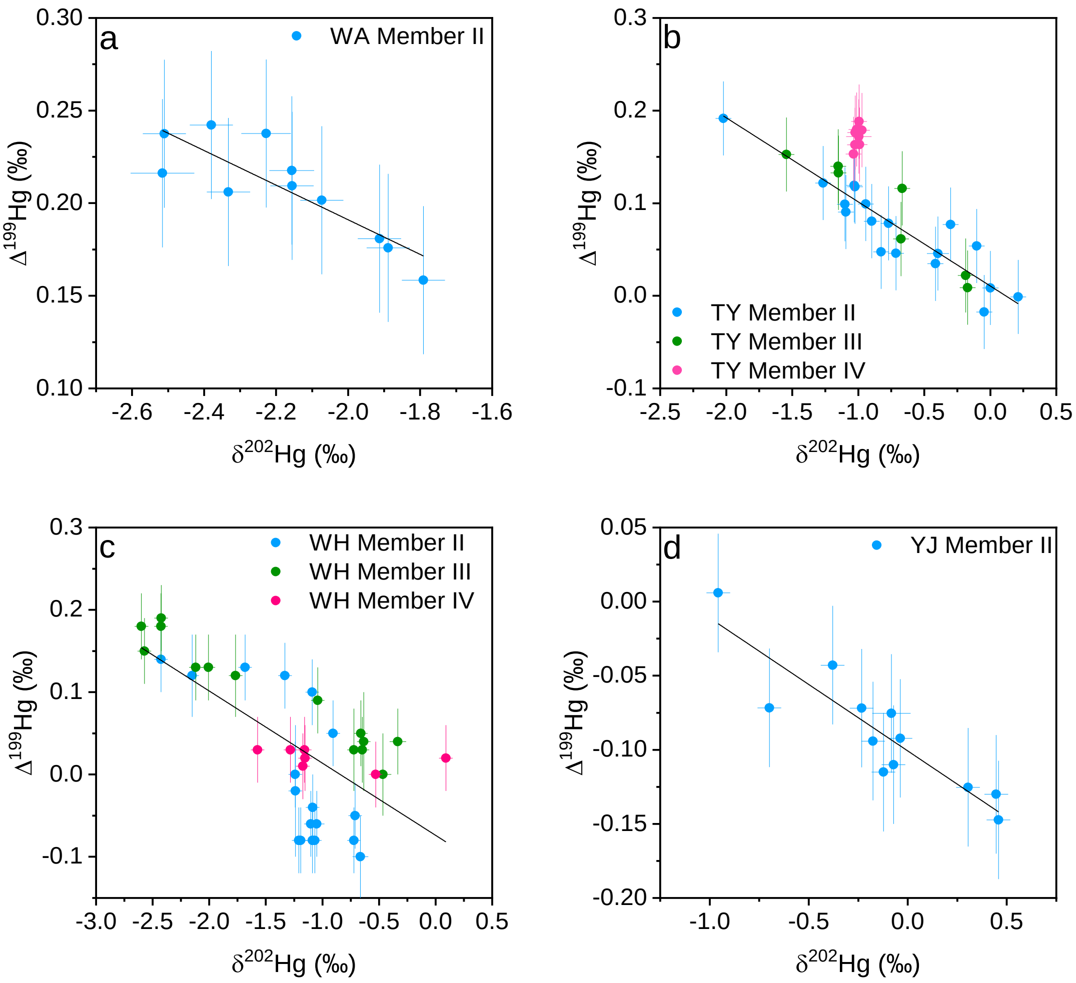

Figure S3. The cross plots of Δ^{200}Hg vs. δ^{202}Hg for all Doushantuo sections. The slope of the linear regression is −0.01 ± 0.00 (1SE, R^2 = 0.46, P = 0).

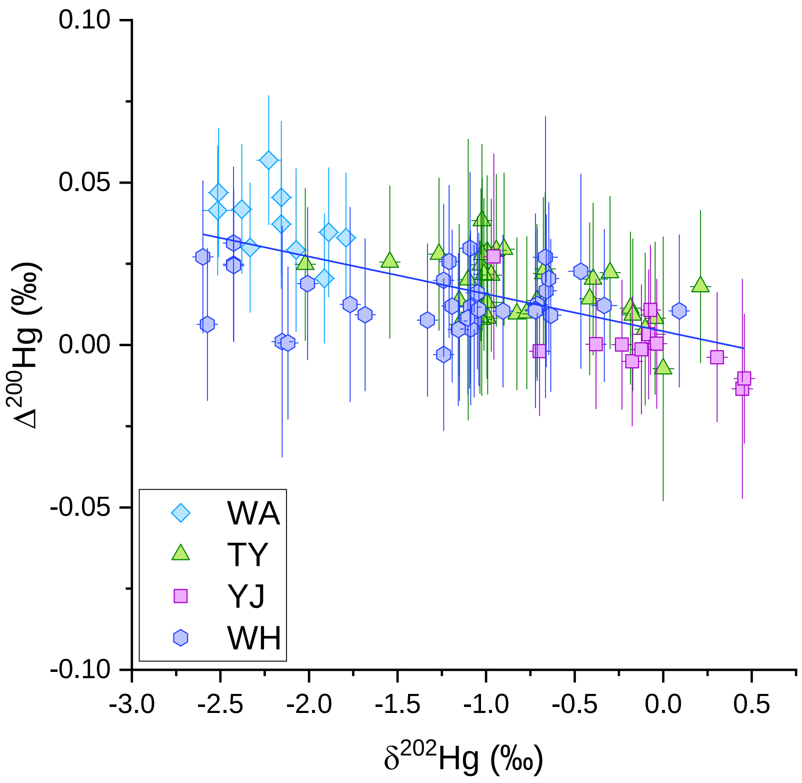

Figure S4. The relationship between Δ^{199}Hg and Δ^{201}Hg for all samples in this study. The blue line is the linear regression with y error only (slope = 1.45 ± 0.03, 1SE, R^2 = 0.96, P = 0), and the red solid line is the linear regression with both x and y errors (York regression, slope = 1.49 ± 0.09, 1SE, R^2 = 0.96, P = 0). The red dash line (slope = 1.60) is the reference line generated by the experimental study of dark abiotic oxidation of Hg(0) by thiol compounds[60]. The black dash-dot line (slope = 1.0) is the typical regression line for atmospheric Hg(II) and Hg(0)[61]. The two reference lines are presented here to demonstrate that the Δ^{199}Hg/Δ^{201}Hg slope of Doushantuo shales is similar to that of the dark oxidation of Hg(0), but significantly different from that of atmospheric Hg.

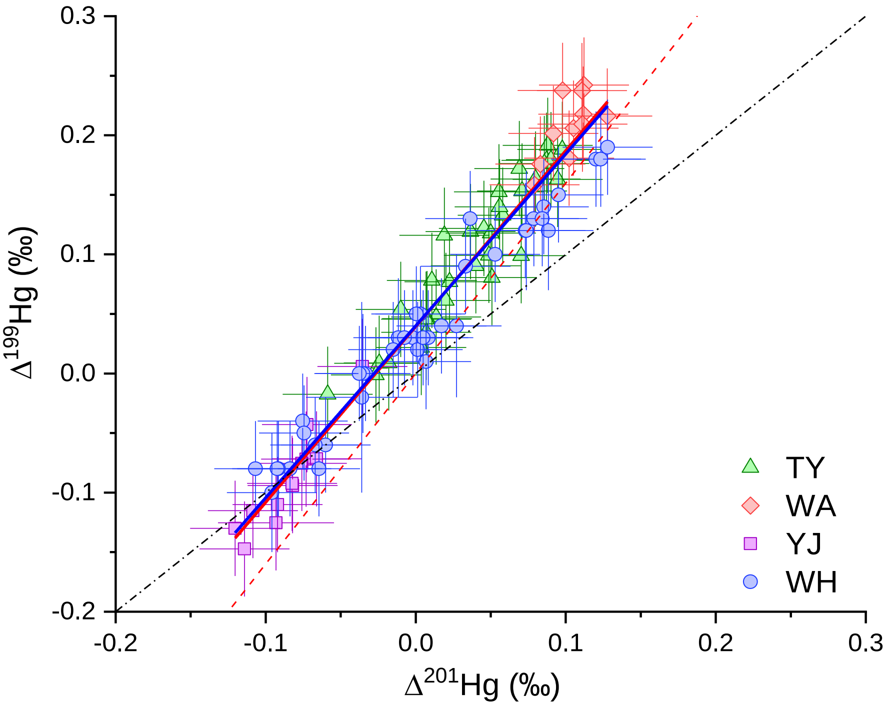

Figure S5. The cross-plots of a) Δ^{199}Hg vs. δ^{202}Hg and b) Δ^{199}Hg vs. Δ^{200}Hg for a compilation of modern samples in the literature (see Text S3 for a complete list of sample types and references). These literature data are plotted in both individual point and in average with 1SD.

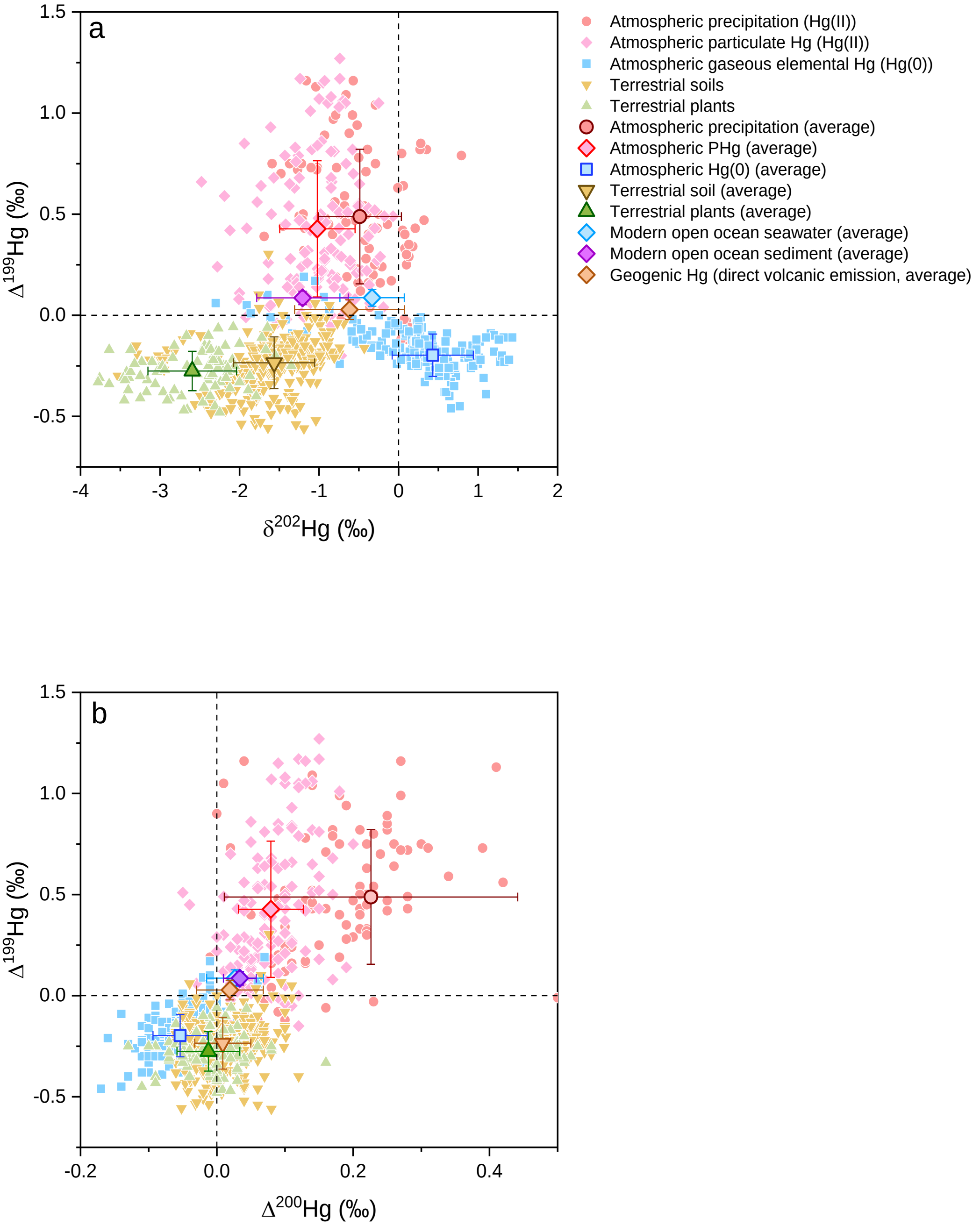

Figure S6. A simplified ocean–atmosphere Hg isotope box-model. The numbers in boxes represent initial Hg reservoir sizes (Mg), the numbers beside the arrows represent Hg fluxes ($Mg{\cdot}a^{-1}$) which are differentiated by processes identifiers (volcanic Hg emission, hydrothermal Hg discharges, photochemical Hg(0) oxidation, photochemical Hg(II) reduction, atmospheric Hg(0) deposition, atmospheric Hg(II) deposition, marine Hg(0) evasion, net lateral water Hg advection, sediment Hg burial).

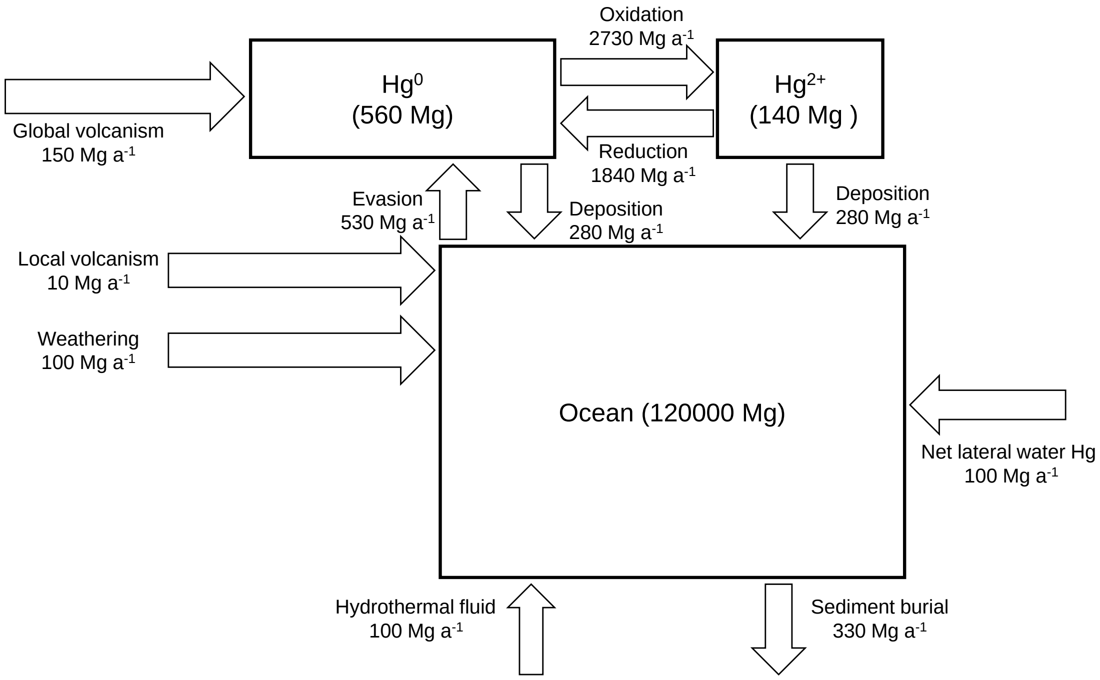

Table S1. Mercury isotope compositions of reference materials and standards.

Sample ID	n	Recovery	δ^{199}Hg (‰)	2σ	δ^{200}Hg (‰)	2σ	δ^{201}Hg (‰)	2σ	δ^{202}Hg (‰)	2σ	Δ^{199}Hg (‰)	2σ	Δ^{200}Hg (‰)	2σ	Δ^{201}Hg (‰)	2σ
NIST 2702	3	98%	-0.22	0.07	-0.38	0.04	-0.61	0.06	-0.77	0.04	-0.03	0.06	0.00	0.02	-0.03	0.04
SBC-1	2	102%	-0.23	0.03	-0.41	0.03	-0.65	0.03	-0.87	0.03	-0.01	0.02	0.03	0.01	0.01	0.01
GBW 07405	5	110%	-0.78	0.08	-0.89	0.05	-1.66	0.09	-1.76	0.13	-0.34	0.06	-0.01	0.03	-0.34	0.06
Column NIST 3133	6	95%	0.00	0.05	-0.01	0.04	-0.01	0.05	-0.02	0.05	0.01	0.04	0.00	0.02	0.00	0.02
Digest NIST 3133	3	108%	-0.01	0.04	-0.03	0.07	-0.03	0.11	-0.04	0.12	0.00	0.02	-0.01	0.02	0.01	0.04

Table S2. Mercury isotope compositions of all samples in this study.

Sample ID	Member	Height (m)	$\delta^{199}Hg$ (‰)	2σ	$\delta^{200}Hg$ (‰)	2σ	$\delta^{201}Hg$ (‰)	2σ	$\delta^{202}Hg$ (‰)	2σ	$\Delta^{199}Hg$ (‰)	2σ	$\Delta^{200}Hg$ (‰)	2σ	$\Delta^{201}Hg$ (‰)	2σ
**WH**																
WH-3.2	II	3.2	-0.32	0.06	-0.60	0.05	-0.97	0.06	-1.24	0.06	0.00	0.05	0.02	0.03	-0.03	0.03
WH-3.7	II	3.7	-0.38	0.06	-0.59	0.05	-1.00	0.06	-1.19	0.06	-0.08	0.05	0.01	0.03	-0.11	0.03
WH-3.7-2	II	3.7	-0.38	0.06	-0.58	0.05	-0.99	0.06	-1.21	0.06	-0.08	0.05	0.03	0.03	-0.08	0.03
WH-4.3	II	4.3	-0.35	0.06	-0.53	0.05	-0.89	0.06	-1.07	0.06	-0.08	0.05	0.01	0.03	-0.09	0.03
WH-4.3-2	II	4.3	-0.35	0.06	-0.53	0.05	-0.88	0.06	-1.09	0.06	-0.08	0.05	0.01	0.03	-0.06	0.03
WH-5.4	II	5.4	-0.33	0.06	-0.62	0.05	-0.96	0.06	-1.24	0.06	-0.02	0.08	0.00	0.03	-0.04	0.04
WH-6.3	II	6.3	-0.32	0.06	-0.54	0.05	-0.89	0.06	-1.09	0.06	-0.04	0.05	0.00	0.03	-0.08	0.03
WH-6.3-2	II	6.3	-0.34	0.06	-0.55	0.05	-0.89	0.06	-1.10	0.06	-0.06	0.05	0.01	0.03	-0.06	0.03
WH-7	II	7	-0.33	0.06	-0.51	0.05	-0.86	0.06	-1.05	0.07	-0.06	0.05	0.02	0.03	-0.07	0.03
WH-9.4	II	9.4	-0.23	0.06	-0.35	0.05	-0.61	0.06	-0.71	0.06	-0.05	0.05	0.01	0.03	-0.07	0.03
WH-11.6	II	11.6	-0.26	0.06	-0.35	0.05	-0.63	0.06	-0.72	0.06	-0.08	0.05	0.01	0.03	-0.09	0.03
WH-13.7	II	13.7	-0.27	0.06	-0.31	0.08	-0.60	0.06	-0.66	0.07	-0.10	0.05	0.03	0.04	-0.10	0.03
WH-21.5	II	21.5	-0.18	0.06	-0.44	0.05	-0.68	0.06	-0.91	0.06	0.05	0.05	0.01	0.03	0.00	0.03
WH-29	II	29	-0.18	0.06	-0.52	0.05	-0.77	0.06	-1.09	0.06	0.10	0.05	0.03	0.03	0.05	0.03
WH-35.1	II	35.1	-0.43	0.06	-1.08	0.06	-1.53	0.06	-2.15	0.06	0.12	0.05	0.00	0.04	0.09	0.03
WH-38.5	II	38.5	-0.47	0.06	-1.19	0.05	-1.74	0.06	-2.43	0.06	0.14	0.05	0.02	0.03	0.09	0.03
WH-47.5	II	47.5	-0.29	0.06	-0.84	0.05	-1.19	0.06	-1.68	0.06	0.13	0.05	0.01	0.03	0.08	0.03
WH-53.4	II	53.4	-0.22	0.06	-0.66	0.05	-0.93	0.06	-1.33	0.06	0.12	0.05	0.01	0.03	0.07	0.03
WH-60.7	III	60.7	-0.12	0.06	-0.31	0.05	-0.45	0.06	-0.63	0.06	0.04	0.06	0.01	0.03	0.03	0.03
WH-63.9	III	63.9	-0.05	0.06	-0.16	0.05	-0.23	0.06	-0.33	0.07	0.04	0.05	0.01	0.03	0.02	0.03
WH-64.5	III	64.5	-0.15	0.06	-0.35	0.05	-0.55	0.06	-0.72	0.06	0.03	0.05	0.01	0.03	-0.01	0.03
WH-65.5	III	65.5	-0.11	0.06	-0.21	0.05	-0.38	0.09	-0.46	0.07	0.00	0.05	0.02	0.03	-0.04	0.03

WH-67.1	III	67.1	-0.12	0.06	-0.32	0.05	-0.50	0.06	-0.66	0.06	0.05	0.05	0.02	0.03	0.00	0.03
WH67.1-2	III	67.1	-0.13	0.06	-0.30	0.05	-0.48	0.06	-0.65	0.06	0.03	0.05	0.02	0.03	0.01	0.03
WH-81.9	III	81.9	-0.17	0.06	-0.51	0.05	-0.75	0.06	-1.04	0.06	0.09	0.05	0.01	0.03	0.03	0.03
WH-85.7	III	85.7	-0.48	0.06	-1.28	0.05	-1.84	0.06	-2.60	0.06	0.18	0.05	0.03	0.03	0.12	0.03
WH-88.3	III	88.3	-0.41	0.06	-1.06	0.05	-1.56	0.06	-2.12	0.06	0.13	0.05	0.00	0.03	0.04	0.03
WH-93	III	93	-0.38	0.06	-0.99	0.05	-1.43	0.06	-2.01	0.06	0.13	0.05	0.02	0.03	0.08	0.03
WH-97.9	III	97.9	-0.50	0.06	-1.29	0.05	-1.84	0.06	-2.57	0.06	0.15	0.05	0.01	0.03	0.10	0.03
WH-102.4	III	102.4	-0.42	0.06	-1.19	0.05	-1.70	0.06	-2.42	0.06	0.19	0.05	0.02	0.03	0.13	0.03
WH-107.4	III	107.4	-0.43	0.06	-1.19	0.05	-1.70	0.06	-2.43	0.06	0.18	0.05	0.03	0.03	0.12	0.03
WH-110.7	III	110.7	-0.32	0.06	-0.88	0.05	-1.26	0.06	-1.77	0.06	0.12	0.05	0.01	0.03	0.07	0.04
WH-114.5	IV	114.5	0.04	0.06	0.06	0.05	0.05	0.06	0.09	0.06	0.02	0.05	0.01	0.03	-0.01	0.04
WH-115.5	IV	115.5	-0.26	0.06	-0.58	0.05	-0.87	0.06	-1.16	0.06	0.03	0.05	0.00	0.03	0.00	0.03
WH-115.5-2	IV	115.5	-0.28	0.06	-0.58	0.05	-0.88	0.06	-1.17	0.06	0.01	0.05	0.01	0.03	0.01	0.03
WH-116.8	IV	116.8	-0.27	0.06	-0.56	0.05	-0.87	0.06	-1.15	0.06	0.02	0.05	0.02	0.03	0.00	0.05
WH-117.8	IV	117.8	-0.14	0.06	-0.27	0.05	-0.44	0.06	-0.53	0.06	0.00	0.05	0.00	0.03	-0.04	0.03
WH-118.9	IV	118.9	-0.30	0.06	-0.63	0.05	-0.97	0.06	-1.28	0.06	0.03	0.05	0.01	0.03	-0.01	0.03
WH-120	IV	120	-0.37	0.06	-0.77	0.05	-1.18	0.06	-1.57	0.06	0.03	0.05	0.03	0.03	0.00	0.03
**TY**																
TY-7.9	II	0.6	-0.19	0.06	-0.54	0.05	-0.78	0.06	-1.09	0.06	0.09	0.05	0.01	0.03	0.04	0.03
TY-8.3	II	1	-0.14	0.06	-0.44	0.05	-0.66	0.06	-0.94	0.06	0.10	0.05	0.03	0.03	0.05	0.03
TY-8.6	II	1.3	-0.20	0.06	-0.61	0.05	-0.91	0.06	-1.27	0.06	0.12	0.05	0.03	0.03	0.05	0.03
TY-9	II	1.7	-0.14	0.06	-0.48	0.05	-0.72	0.06	-1.02	0.06	0.12	0.05	0.04	0.03	0.05	0.03
TY-9.8	II	2.5	-0.14	0.06	-0.49	0.05	-0.74	0.06	-1.03	0.06	0.12	0.05	0.02	0.03	0.04	0.03
TY-10.2	II	2.9	-0.15	0.06	-0.42	0.05	-0.62	0.06	-0.90	0.06	0.08	0.05	0.03	0.03	0.05	0.03
TY-10.7	II	3.4	-0.18	0.06	-0.53	0.07	-0.76	0.06	-1.10	0.06	0.10	0.05	0.02	0.04	0.07	0.03
TY-11.3	II	4	-0.16	0.06	-0.41	0.05	-0.61	0.06	-0.83	0.06	0.05	0.05	0.01	0.03	0.01	0.03

TY-11.8	II	4.5	-0.13	0.06	-0.35	0.05	-0.53	0.09	-0.71	0.06	0.05	0.05	0.01	0.03	0.01	0.04
TY-11.8-2	II	4.5	-0.12	0.06	-0.38	0.05	-0.57	0.06	-0.77	0.06	0.08	0.05	0.01	0.03	0.01	0.03
TY-13.3	II	0.3	-0.03	0.06	-0.01	0.05	-0.09	0.06	-0.05	0.06	-0.02	0.05	0.01	0.03	-0.06	0.03
TY-14	II	1	0.00	0.06	-0.13	0.05	-0.20	0.06	-0.30	0.06	0.08	0.05	0.02	0.03	0.02	0.03
TY-15.5	II	2.5	-0.05	0.06	-0.18	0.08	-0.29	0.06	-0.40	0.08	0.05	0.05	0.02	0.03	0.01	0.03
TY-16.5	II	3.5	0.02	0.06	-0.05	0.05	-0.09	0.06	-0.10	0.06	0.05	0.05	0.00	0.03	-0.01	0.03
TY-17.5	II	4.5	-0.07	0.06	-0.19	0.05	-0.31	0.06	-0.42	0.06	0.03	0.05	0.01	0.03	0.01	0.03
TY-18.8	II	5.8	0.01	0.06	-0.01	0.07	-0.02	0.06	0.00	0.06	0.01	0.05	-0.01	0.04	-0.02	0.03
TY-21.3	II	8.3	0.05	0.06	0.12	0.05	0.13	0.06	0.21	0.06	0.00	0.05	0.02	0.03	-0.03	0.03
TY-30.4	II	17.4	-0.32	0.06	-0.99	0.05	-1.43	0.06	-2.02	0.06	0.19	0.05	0.02	0.03	0.09	0.03
TY-34.8	III	21.8	-0.02	0.06	-0.08	0.05	-0.14	0.06	-0.19	0.06	0.02	0.05	0.01	0.03	0.00	0.03
TY-35.9	III	22.9	-0.24	0.06	-0.75	0.05	-1.11	0.06	-1.54	0.06	0.15	0.05	0.03	0.03	0.06	0.03
TY-36.3	III	23.3	-0.11	0.06	-0.32	0.06	-0.49	0.06	-0.68	0.06	0.06	0.05	0.02	0.03	0.02	0.03
TY-36.3-2	III	23.3	-0.05	0.06	-0.31	0.06	-0.48	0.06	-0.67	0.06	0.12	0.05	0.02	0.03	0.02	0.03
TY-38.5	III	25.5	-0.16	0.06	-0.57	0.06	-0.81	0.06	-1.15	0.06	0.13	0.05	0.01	0.03	0.06	0.03
TY-38.8	III	25.8	-0.15	0.06	-0.57	0.06	-0.81	0.06	-1.15	0.06	0.14	0.05	0.01	0.03	0.06	0.03
TY-40	III	27	-0.03	0.06	-0.08	0.06	-0.15	0.06	-0.17	0.06	0.01	0.05	0.01	0.03	-0.02	0.03
TY-48	IV	35	-0.06	0.06	-0.47	0.06	-0.65	0.06	-0.99	0.06	0.19	0.05	0.03	0.03	0.10	0.03
TY-49	IV	36	-0.07	0.06	-0.47	0.06	-0.64	0.06	-0.97	0.06	0.18	0.05	0.02	0.03	0.09	0.03
TY-50	IV	37	-0.08	0.06	-0.49	0.06	-0.68	0.06	-1.02	0.06	0.18	0.05	0.03	0.03	0.09	0.03
TY-50-2	IV	37	-0.08	0.06	-0.49	0.06	-0.67	0.06	-1.01	0.06	0.18	0.05	0.02	0.03	0.09	0.03
TY-52	IV	39	-0.09	0.06	-0.49	0.06	-0.67	0.06	-0.99	0.06	0.16	0.05	0.01	0.03	0.08	0.03
TY-55	IV	42	-0.10	0.06	-0.51	0.06	-0.68	0.06	-1.02	0.06	0.16	0.05	0.01	0.03	0.09	0.03
TY-57	IV	44	-0.08	0.06	-0.49	0.06	-0.68	0.07	-1.00	0.10	0.17	0.05	0.01	0.03	0.07	0.03
TY-59	IV	46	-0.11	0.06	-0.51	0.05	-0.71	0.06	-1.03	0.06	0.15	0.05	0.01	0.03	0.07	0.03

YJ

YJ-5.6	II	5.6	-0.02	0.06	0.21	0.06	0.22	0.06	0.45	0.06	-0.13	0.05	-0.01	0.03	-0.12	0.04
YJ-5.6-2	II	5.6	-0.03	0.06	0.22	0.05	0.23	0.06	0.46	0.06	-0.15	0.05	-0.01	0.03	-0.11	0.04
YJ-5.7	II	5.7	-0.05	0.06	0.15	0.05	0.14	0.06	0.30	0.06	-0.13	0.05	0.00	0.03	-0.09	0.04
YJ-6.3	II	6.3	-0.13	0.06	-0.12	0.05	-0.25	0.06	-0.23	0.06	-0.07	0.05	0.00	0.03	-0.07	0.03
YJ-6.8	II	6.8	-0.10	0.06	-0.04	0.07	-0.14	0.07	-0.08	0.10	-0.08	0.05	0.00	0.03	-0.08	0.03
YJ-6.9	II	6.9	-0.14	0.06	-0.09	0.05	-0.21	0.06	-0.18	0.06	-0.09	0.05	-0.01	0.03	-0.08	0.03
YJ-6.95	II	6.95	-0.13	0.06	-0.03	0.05	-0.15	0.06	-0.07	0.06	-0.11	0.05	0.01	0.03	-0.09	0.03
YJ-7.2	II	7.2	-0.10	0.06	-0.02	0.05	-0.11	0.06	-0.04	0.06	-0.09	0.05	0.00	0.03	-0.08	0.03
YJ-7.4	II	7.4	-0.15	0.06	-0.06	0.05	-0.20	0.06	-0.12	0.06	-0.12	0.05	0.00	0.03	-0.11	0.03
YJ-7.6	II	7.6	-0.14	0.06	-0.19	0.05	-0.36	0.06	-0.38	0.06	-0.04	0.05	0.00	0.03	-0.07	0.03
YJ-9.1	II	9.1	-0.25	0.06	-0.35	0.05	-0.59	0.06	-0.70	0.06	-0.07	0.05	0.00	0.03	-0.07	0.03
YJ-9.5	II	9.5	-0.24	0.06	-0.45	0.05	-0.76	0.06	-0.96	0.06	0.01	0.05	0.03	0.03	-0.04	0.03
**WA**																
WA-6	II	9.9	-0.36	0.06	-1.15	0.05	-1.68	0.06	-2.38	0.06	0.24	0.05	0.04	0.03	0.11	0.03
WA-10	II	10.3	-0.40	0.06	-1.22	0.05	-1.78	0.06	-2.51	0.06	0.24	0.05	0.05	0.03	0.11	0.03
WA-12	II	10.5	-0.32	0.06	-1.01	0.05	-1.47	0.06	-2.07	0.06	0.20	0.05	0.03	0.03	0.09	0.03
WA-15	II	10.9	-0.38	0.06	-1.14	0.05	-1.65	0.06	-2.33	0.06	0.21	0.05	0.03	0.03	0.11	0.03
WA-19	II	11.6	-0.42	0.06	-1.22	0.05	-1.76	0.07	-2.52	0.09	0.22	0.05	0.04	0.03	0.13	0.03
WA-22	II	12.1	-0.32	0.06	-1.06	0.05	-1.58	0.07	-2.23	0.07	0.24	0.05	0.06	0.03	0.10	0.03
WA-26	II	12.6	-0.33	0.06	-1.04	0.05	-1.51	0.06	-2.16	0.06	0.22	0.05	0.05	0.03	0.11	0.03
WA-26-2	II	12.6	-0.33	0.06	-1.05	0.05	-1.51	0.06	-2.16	0.06	0.21	0.05	0.04	0.03	0.11	0.04
WA-29	II	12.9	-0.29	0.06	-0.87	0.05	-1.27	0.06	-1.79	0.06	0.16	0.05	0.03	0.03	0.08	0.03
WA-33	II	13.5	-0.30	0.06	-0.94	0.05	-1.34	0.06	-1.91	0.06	0.18	0.05	0.02	0.03	0.10	0.03
WA-37	II	14.2	-0.30	0.06	-0.91	0.05	-1.34	0.06	-1.89	0.06	0.18	0.05	0.03	0.03	0.08	0.03

Note: 2σ is either 2SE of replicates of each sample, or 2SD of UM-Almaden, whichever is higher. Sample IDs with "-2" are procedural replicates

Table S3. Raw and normalized Hg concentrations (Hg/TOC, Hg/S, Hg/Al, Hg/Fe). TOC, S_{pyrite}, Al and total Fe data are from ref [5].

Sample No.	Member	Strat.Height (m)	Hg conc (ppb)	TOC (wt %)	Hg/TOC	S_{pyrite} (wt %)	Hg/S	Al (wt %)	Hg/Al	Total Fe (wt %)	Hg/Fe
**WH**											
WH-2.4	II	2.4	--	0.7	--	0.2	--	10.4	--	2.2	--
WH-2.7	II	2.7	80.4	0.6	132.0	0.2	476.8	8.7	9.2	1.7	46.4
WH-3	II	3.0	142.6	0.8	172.7	0.6	225.2	8.4	16.9	1.9	74.1
WH-3.2	II	3.2	286.9	0.9	306.3	1.4	204.2	8.6	33.4	2.6	111.8
WH-3.5	II	3.5	104.4	0.8	129.7	0.2	492.0	9.4	11.2	2.1	50.7
WH-3.7	II	3.7	325.6	1.3	259.7	2.0	164.5	9.0	36.1	3.2	103.1
WH-4	II	4.0	123.2	0.9	135.1	0.9	143.8	8.7	14.2	2.3	54.0
WH-4.3	II	4.3	306.8	1.2	258.0	2.2	139.6	9.4	32.5	3.9	78.6
WH-4.6	II	4.6	196.7	0.8	256.5	1.3	152.9	6.6	29.7	3.2	61.9
WH-4.8	II	4.8	184.5	1.2	159.1	0.7	282.2	8.5	21.7	2.6	69.8
WH-5.0	II	5.0	157.4	0.5	337.3	1.5	108.0	3.4	45.7	5.3	29.9
WH-5.4	II	5.4	244.3	1.3	189.5	1.8	134.6	8.5	28.8	3.1	77.7
WH-5.6	II	5.6	251.7	1.1	225.1	1.7	152.1	7.0	35.8	4.0	63.6
WH-5.8	II	5.8	287.5	0.8	363.3	1.8	162.7	6.6	43.3	3.5	82.3
WH-6.0	II	6.0	471.3	1.4	345.7	3.0	158.6	10.0	47.2	3.8	124.3
WH-6.3	II	6.3	425.1	1.4	305.4	3.1	139.3	10.1	42.0	3.8	110.7
WH-6.5	II	6.5	190.3	1.2	165.2	0.0	4992.8	9.3	20.4	2.5	77.3
WH-6.8	II	6.8	222.1	1.2	185.9	1.5	143.6	9.6	23.1	2.9	77.1
WH-7.0	II	7.0	326.8	1.2	283.7	2.2	146.1	9.2	35.7	3.4	95.3
WH-7.3	II	7.3	275.9	1.3	215.4	1.7	159.5	8.2	33.5	2.9	94.5
WH-8.6	II	8.6	285.6	1.2	243.9	2.5	114.0	8.6	33.0	3.8	76.0
WH-9.4	II	9.4	294.0	0.8	372.9	2.3	127.5	7.8	37.5	3.4	85.3

WH-9.7	II	9.7	290.1	1.3	228.7	2.2	132.3	7.9	36.5	3.0	97.5
WH-11.6	II	11.6	335.0	1.3	267.9	2.2	153.9	6.8	49.4	3.0	112.4
WH-13.7	II	13.7	176.9	0.6	295.3	1.8	98.9	4.2	41.9	2.5	71.6
WH-21.5	II	21.5	231.6	0.9	249.1	--	118.9	6.0	38.6	3.0	76.8
WH-24.1	II	24.1	307.6	1.0	299.9	2.8	109.3	5.6	54.7	3.7	83.1
WH-26.0	II	26.0	284.2	1.9	149.3	2.8	101.5	6.1	46.7	3.3	87.2
WH-29.0	II	29.0	309.9	1.6	193.5	3.1	100.8	6.4	48.6	3.7	84.1
WH-32.4	II	32.4	196.1	1.3	146.8	2.8	71.1	3.8	52.2	2.9	68.4
WH-35.1	II	35.1	200.4	2.2	92.7	2.5	79.3	3.7	53.6	2.6	78.2
WH-38.5	II	38.5	446.6	1.4	315.4	2.6	169.5	6.4	69.9	3.9	114.6
WH-41.1	II	41.1	--	2.5	--	3.6	--	4.9	--	3.6	--
WH-44.3	II	44.3	--	1.7	--	1.7	--	2.4	--	1.9	--
WH-47.5	II	47.5	880.7	5.7	155.0	3.2	275.7	3.7	237.4	2.4	365.2
WH-50.1	II	50.1	689.6	3.6	193.2	2.6	263.1	3.5	199.6	2.6	265.4
WH-53.4	II	53.4	714.2	3.3	213.7	3.3	215.8	5.4	132.7	3.6	197.2
WH-54.9	II	54.9	490.8	1.6	309.4	4.0	123.3	4.8	103.2	3.1	158.6
WH-56.3	II	56.3	310.5	0.9	341.8	4.5	69.5	4.3	73.0	2.8	112.3
WH-57.3	II	57.3	302.8	0.7	449.0	1.7	179.6	6.2	48.8	2.4	124.6
WH-60.7	III	60.7	305.7	1.8	172.2	0.9	326.3	3.0	103.6	1.3	227.6
WH-61.1	III	61.1	277.7	0.3	961.7	0.5	598.9	2.1	134.8	1.2	239.5
WH-62.4	III	62.4	31.7	0.3	117.0	0.1	221.4	1.7	18.5	1.0	31.6
WH-63.1	III	63.1	--	0.7	--	1.0	--	3.5	--	1.4	--
WH-63.7	III	63.7	--	2.1	--	0.6	--	6.1	--	1.6	--
WH-63.9	III	63.9	496.5	1.0	478.7	0.6	859.0	3.8	130.4	1.0	476.3
WH-64.5	III	64.5	360.1	2.1	170.5	2.0	181.0	7.9	45.4	2.5	143.3
WH-65.5	III	65.5	522.8	0.6	896.1	0.9	601.8	8.2	63.4	1.5	342.5

WH-66.3	III	66.3	--	1.6	--	0.1	--	6.9	--	1.9	--
WH-66.8	III	66.8	--	2.1	--	0.5	--	8.5	--	2.4	--
WH-67.1	III	67.1	544.6	2.7	199.7	0.0	18746.4	8.8	62.1	2.3	231.8
WH-67.4	III	67.4	--	2.2	--	0.0	--	8.5	--	2.4	--
WH-81.9	III	81.9	296.4	1.0	291.2	2.3	128.1	7.1	42.0	4.0	74.4
WH-82.8	III	82.8	230.5	0.6	381.6	2.4	96.8	5.6	41.5	2.8	82.4
WH-83.7	III	83.7	292.3	1.5	194.7	2.8	105.1	8.5	34.6	5.0	58.4
WH-84.5	III	84.5	291.3	1.2	249.0	2.8	104.3	4.9	59.6	2.8	104.3
WH-85.7	III	85.7	661.1	3.1	214.2	3.7	178.5	7.9	83.6	4.1	161.8
WH-86.6	III	86.6	329.4	1.3	257.0	3.0	110.5	6.2	52.8	3.4	97.6
WH-87.1	III	87.1	174.2	1.2	147.8	2.1	84.7	3.3	52.8	2.4	73.3
WH-88.3	III	88.3	1240.5	3.6	348.9	2.7	461.6	6.7	185.0	3.8	326.0
WH-89.2	III	89.2	669.7	3.5	189.8	4.1	162.3	7.4	90.2	4.2	158.4
WH-90.7	III	90.7	431.9	1.6	276.4	2.9	149.9	4.7	91.3	3.0	143.6
WH-91.5	III	91.5	349.0	2.0	178.8	2.8	123.0	4.4	79.0	3.1	110.9
WH-93.0	III	93.0	367.4	2.7	135.5	2.7	138.4	4.4	83.5	3.1	117.0
WH-94.0	III	94.0	131.2	3.4	38.6	0.8	171.2	1.5	86.7	1.2	105.6
WH-95.6	III	95.6	784.6	3.4	230.2	4.1	191.1	4.7	167.7	4.4	177.6
WH-96.3	III	96.3	546.7	2.0	268.9	3.7	147.6	5.2	105.8	3.9	139.5
WH-97.9	III	97.9	627.9	2.7	233.1	3.7	169.3	5.9	106.2	3.5	178.2
WH-99.6	III	99.6	276.0	2.9	94.7	1.9	141.7	3.1	89.4	2.2	126.8
WH-100.4	III	100.4	376.1	2.9	128.6	1.8	214.4	3.6	104.3	2.2	169.4
WH-101.2	III	101.2	290.0	0.9	310.8	1.5	188.5	2.0	141.6	1.7	167.5
WH-102.4	III	102.4	853.4	5.9	144.4	3.2	270.7	3.9	221.3	3.0	280.1
WH-103.3	III	103.3	390.0	5.4	71.8	1.6	243.2	3.1	127.9	1.8	213.3
WH-104.1	III	104.1	756.1	4.8	158.4	3.0	254.7	4.3	175.5	3.4	221.5

WH-105.3	III	105.3	671.8	5.0	134.6	2.4	277.7	4.6	145.5	3.2	206.9
WH-106.4	III	106.4	473.4	4.3	110.0	2.6	184.0	5.2	90.8	3.1	153.3
WH-107.4	III	107.4	624.9	2.9	218.4	3.3	188.6	5.3	117.5	3.6	171.6
WH-108.3	III	108.3	139.7	0.5	269.4	1.7	80.8	5.2	27.0	2.0	69.3
WH-110.7	III	110.7	348.4	0.6	540.4	1.0	363.1	1.2	296.3	1.1	320.6
WH-114.5	IV	114.5	221.2	1.1	194.3	0.8	283.5	2.8	78.5	1.0	221.4
WH-115.3	IV	115.3	961.9	3.8	255.5	1.6	600.9	7.1	135.7	2.5	389.4
WH-115.5	IV	115.5	536.5	1.5	369.8	1.9	288.6	7.4	72.6	2.5	218.8
WH-115.9	IV	115.9	1055.0	2.9	364.0	1.8	592.4	8.0	132.2	2.4	441.2
WH-116.3	IV	116.3	1050.1	1.9	543.5	1.2	885.0	7.1	147.7	1.9	553.3
WH-116.8	IV	116.8	852.8	5.0	170.1	1.3	641.6	5.7	150.5	1.8	473.2
WH-117.4	IV	117.4	2475.8	5.2	472.1	2.8	883.9	5.2	471.8	3.2	776.2
WH-117.8	IV	117.8	2572.4	8.5	301.1	2.5	1045.1	5.2	496.6	3.4	749.1
WH-118.3	IV	118.3	2791.0	9.3	299.2	2.3	1216.7	3.6	777.3	2.6	1060.8
WH-118.9	IV	118.9	1261.0	7.0	180.7	1.2	1017.8	2.0	636.5	1.3	937.0
WH-119.5	IV	119.5	1289.8	7.1	182.8	1.2	1036.8	2.4	542.3	1.5	887.3
WH-120.0	IV	120.0	445.9	4.1	108.8	0.6	799.0	2.3	197.8	0.8	527.7
**TY**											
TY09-7.6	II	0.3	298.7	1.2	251.3	0.0	9277.9	7.4	40.1	0.8	354.3
TY09-7.9	II	0.6	340.5	1.3	272.3	0.2	1868.4	7.7	44.3	0.7	457.7
TY09-8.0	II	0.7	330.0	1.3	257.4	0.0	7579.0	8.1	40.6	0.7	444.2
TY09-8.3	II	1.0	555.1	1.9	291.1	0.0	80870.1	8.4	66.1	0.6	869.0
TY09-8.6	II	1.3	496.9	1.5	335.7	0.0	24579.4	7.9	63.0	0.7	688.7
TY09-8.8	II	1.5	467.6	1.6	289.7	0.1	5236.3	8.7	54.0	0.6	732.2
TY09-9.0	II	1.7	598.4	2.1	284.4	0.0	13674.3	8.2	73.3	0.6	1027.5

TY09-9.4	II	2.1	848.7	1.9	436.4	0.0	23989.6	8.6	99.0	0.6	1436.3
TY09-9.8	II	2.5	601.8	1.6	385.3	0.0	18481.8	9.5	63.6	0.8	781.8
TY09-10.0	II	2.7	981.2	1.9	517.6	0.0	51903.2	8.3	118.8	0.6	1688.2
TY09-10.2	II	2.9	1098.3	2.4	459.1	0.1	8180.4	9.8	111.7	0.7	1567.6
TY09-10.4	II	3.1	887.7	2.2	407.8	0.1	9863.3	7.7	115.5	0.6	1407.9
TY09-10.7	II	3.4	439.1	1.5	283.5	0.0	9263.2	8.9	49.3	0.7	673.7
TY09-11.0	II	3.7	712.7	2.1	337.9	0.5	1377.8	5.5	128.8	0.4	1717.1
TY09-11.3	II	4.0	931.6	2.1	434.5	0.1	9401.5	7.3	127.0	0.6	1618.5
TY09-11.5	II	4.2	1105.5	2.4	469.1	0.1	9988.6	7.1	155.5	0.5	2443.0
TY09-11.8	II	4.5	1083.1	2.4	448.2	0.1	19656.2	7.2	151.0	0.5	2252.6
TY09-12.0	II	4.7	714.4	2.2	318.3	0.0	40869.0	7.6	94.4	0.5	1342.7
TY09-13.3	II	0.3	1738.0	0.4	4759.2	0.8	2249.2	8.3	209.2	1.5	1171.8
TY09-13.8	II	0.8	362.5	1.3	272.4	0.6	657.9	9.0	40.3	0.8	444.1
TY09-14.0	II	1.0	426.0	2.0	210.0	0.2	2577.1	8.8	48.6	0.7	582.6
TY09-15.1	II	2.1	538.0	1.7	312.4	0.4	1246.8	19.4	27.7	1.3	416.5
TY09-15.5	II	2.5	959.4	2.5	389.0	0.5	2026.3	7.7	124.4	0.6	1482.0
TY09-16.3	II	3.3	842.8	2.6	326.4	0.7	1124.7	8.8	95.3	0.7	1127.9
TY09-16.5	II	3.5	896.4	3.2	278.9	1.6	546.7	8.9	101.2	2.1	422.0
TY09-17.5	II	4.5	360.3	1.9	192.9	0.5	799.0	8.8	41.0	1.5	243.7
TY09-18.8	II	5.8	765.5	2.6	296.3	0.5	1668.4	6.7	114.4	0.8	920.0
TY09-19.7	II	6.7	805.9	2.8	292.3	0.5	1671.9	7.2	112.6	0.9	871.7
TY09-21.3	II	8.3	3590.8	4.5	794.8	1.0	3429.8	6.1	591.6	0.6	5750.4
TY09-22.3	II	9.3	819.1	1.0	798.3	1.7	484.4	5.5	148.0	1.0	844.2
TY09-30.4	III	17.4	258.9	6.7	38.8	0.2	1101.7	10.1	25.6	1.0	260.6
TY09-34.8	III	21.8	2072.1	2.4	864.1	0.2	13206.9	5.7	361.7	0.8	2600.3
TY09-35.4	III	22.4	3571.7	8.8	406.6	0.2	21498.6	5.5	643.8	0.4	8808.6

TY09-35.9	III	22.9	3831.5	9.7	394.5	0.1	26140.0	4.7	811.2	0.3	11533.7
TY09-36.3	III	23.3	3155.2	11.7	270.1	0.2	20939.9	4.2	760.2	0.3	11316.8
TY09-38.5	III	25.5	3442.8	7.1	484.5	0.1	23784.6	3.6	969.5	0.3	10356.4
TY09-38.8	III	25.8	3316.0	4.4	751.0	0.2	22072.1	5.0	669.2	0.4	8600.1
TY09-39.0	III	26.0	1855.2	5.6	328.6	0.1	13981.5	2.7	689.6	0.3	5554.9
TY09-40.0	III	27.0	2322.5	2.5	936.1	0.1	17492.1	3.2	730.1	0.3	8541.7
TY09-47.0	IV	34.0		5.7	0.0	0.1		2.3		0.3	
TY09-48.0	IV	35.0	3122.2	4.6	678.7	0.2	14720.6	7.5	415.9	1.0	3117.0
TY09-49.0	IV	36.0	3200.8	4.2	757.6	0.1	26919.0	9.1	350.8	1.2	2706.7
TY09-50.0	IV	37.0	3411.6	4.9	696.8	0.1	60703.0	8.2	417.7	0.5	6212.5
TY09-51.0	IV	38.0	2068.9	5.8	354.6	0.1	16800.6	6.4	321.8	0.6	3750.1
TY09-52.0	IV	39.0	927.7	7.0	132.2	0.1	7003.7	2.3	411.1	0.3	3528.1
TY09-53.0	IV	40.0	2050.4	5.7	359.2	0.1	16506.4	5.8	356.5	0.5	4191.4
TY09-55.0	IV	42.0	1483.5	7.8	189.7	0.1	10766.1	4.5	330.7	1.0	1543.5
TY09-57.0	IV	44.0	3725.8	2.9	1299.0	0.1	27847.7	12.4	301.4	2.9	1267.1
TY09-59.0	IV	46.0	1915.4	2.4	787.3	0.1	14989.7	4.0	480.3	0.8	2526.9
**WA**											
WA-6	II	9.9	719.1	0.2	3595.3	1.1	630.8	4.0	179.8	1.5	479.4
WA-10	II	10.3	580.2	0.2	3053.6	1.2	500.2	0.9	644.6	1.2	483.5
WA-12	II	10.5	643.4	0.1	4949.5	1.8	357.5	0.7	919.2	1.6	402.1
WA-15	II	10.9	423.7	0.1	3531.0	1.2	362.2	0.5	847.4	1.2	353.1
WA-19	II	11.6	716.0	0.3	2753.8	1.7	413.9	2.3	311.3	2.0	358.0
WA-22	II	12.1	649.5	0.3	2597.9	1.6	405.9	2.1	309.3	1.7	382.0
WA-26	II	12.6	1431.9	0.5	2807.6	1.8	809.0	8.3	172.5	2.2	650.8
WA-29	II	12.9	870.1	0.3	3000.2	1.5	565.0	2.0	435.0	1.8	483.4

WA-33	II	13.5	999.2	0.4	2855.0	1.3	762.8	3.6	277.6	1.7	587.8
WA-37	II	14.2	616.7	0.3	2126.5	1.7	360.6	2.3	268.1	2.0	308.3
**YJ**											
YJ-5.6	II	5.6	3250.7	1.7	1864.2	0.5	7223.8	9.9	328.4	0.7	4643.9
YJ-5.7	II	5.7	1443.9	1.5	983.0	1.9	768.0	9.6	150.4	2.4	601.6
YJ-5.9	II	5.9		1.6	0.0			9.4		1.9	
YJ-6.3	II	6.3	659.6	2.4	269.8	2.0	338.3	8.8	75.0	2.7	244.3
YJ-6.4	II	6.4	580.1	2.7	216.9	2.4	237.7	8.7	66.7	3.1	187.1
YJ-6.8	II	6.8	370.8	3.0	123.0	2.6	143.2	7.6	48.8	2.9	127.9
YJ-6.9	II	6.9	226.0	2.1	108.5	3.2	70.9	8.6	26.3	4.2	53.8
YJ-6.95	II	7.0	569.0								
YJ-7.2	II	7.2	359.0	2.7	133.0	4.1	88.6	7.1	50.6	4.2	85.5
YJ-7.4	II	7.4	433.1	3.6	119.5	4.1	106.4	7.4	58.5	4.5	96.2
YJ-7.6	II	7.6	419.8	4.1	103.5	0.1	3229.2	8.5	49.4	0.6	699.7
YJ-9.1	II	9.1	42.7	0.6	71.5	0.0	4274.8	6.2	6.9	0.6	71.2
YJ-9.5	II	9.5	59.9	0.8	73.3	0.1	1197.9	6.2	9.7	0.7	85.6

Supplementary References:

1. Shen, J. *et al.* Sedimentary host phases of mercury (Hg) and implications for use of Hg as a volcanic proxy. *Earth Planet. Sci. Lett.* **543**, 116333 (2020).
2. Chen, D., Ren, D., Deng, C., Tian, Z. & Yin, R. Mercury loss and isotope fractionation during high-pressure and high-temperature processing of sediments: Implication for the behaviors of mercury during metamorphism. *Geochim. Cosmochim. Acta* **334**, 231–240 (2022).
3. Liu, Z., Tian, H., Yin, R., Chen, D. & Gai, H. Mercury loss and isotope fractionation during thermal maturation of organic-rich mudrocks. *Chem. Geol.* **612**, 121144 (2022).
4. Deng, C. *et al.* Mercury isotopic compositions of the Precambrian rocks and implications for tracing mercury cycling in Earth's interior. *Precambrian Res.* **373**, 106646 (2022).
5. Sahoo, S. K. *et al.* Oceanic oxygenation events in the anoxic Ediacaran ocean. *Geobiology* **14**, 457–468 (2016).
6. Ostrander, C. M. *et al.* Multiple negative molybdenum isotope excursions in the Doushantuo Formation (South China) fingerprint complex redox-related processes in the Ediacaran Nanhua Basin. *Geochim. Cosmochim. Acta* **261**, 191–209 (2019).
7. Ostrander, C. M. *et al.* Thallium isotope ratios in shales from South China and northwestern Canada suggest widespread O2 accumulation in marine bottom waters was an uncommon occurrence during the Ediacaran Period. *Chem. Geol.* **557**, 119856 (2020).
8. Xu, D. *et al.* Chromium isotope evidence for oxygenation events in the Ediacaran ocean. *Geochim. Cosmochim. Acta* **323**, 258–275 (2022).
9. Jiskra, M., Wiederhold, J. G., Skyllberg, U., Kronberg, R.-M. & Kretzschmar, R. Source tracing of natural organic matter bound mercury in boreal forest runoff with mercury stable isotopes. *Environ. Sci. Process. Impacts* **19**, 1235–1248 (2017).
10. Biswas, A., Blum, J. D., Bergquist, B. A., Keeler, G. J. & Xie, Z. Natural mercury isotope variation in coal deposits and organic soils. *Environ. Sci. Technol.* **42**, 8303–8309 (2008).
11. Demers, J. D., Blum, J. D. & Zak, D. R. Mercury isotopes in a forested ecosystem: Implications for air-surface exchange dynamics and the global mercury cycle. *Glob. Biogeochem. Cycles* **27**, 222–238 (2013).
12. Jiskra, M. *et al.* Mercury deposition and re-emission pathways in boreal forest soils investigated with Hg isotope signatures. *Environ. Sci. Technol.* **49**, 7188–7196 (2015).
13. Enrico, M. *et al.* Atmospheric mercury transfer to peat bogs dominated by gaseous elemental mercury dry deposition. *Environ. Sci. Technol.* **50**, 2405–2412 (2016).
14. Enrico, M. *et al.* Holocene atmospheric mercury levels reconstructed from peat bog mercury stable isotopes. *Environ. Sci. Technol.* **51**, 5899–5906 (2017).
15. Zheng, W., Obrist, D., Weis, D. & Bergquist, B. A. Mercury isotope compositions across North American forests. *Glob. Biogeochem. Cycles* **30**, 1475–1492 (2016).
16. Wang, X. *et al.* Using mercury isotopes to understand mercury accumulation in the montane forest floor of the Eastern Tibetan Plateau. *Environ. Sci. Technol.* **51**, 801–809 (2017).
17. Wang, X. *et al.* Global warming accelerates uptake of atmospheric mercury in regions experiencing glacier retreat. *Proc. Natl. Acad. Sci. U. S. A.* **117**, 2049–2055 (2020).
18. Obrist, D. *et al.* Tundra uptake of atmospheric elemental mercury drives Arctic mercury pollution. *Nature* **547**, 201–204 (2017).
19. Yuan, W. *et al.* Stable Isotope Evidence Shows Re-emission of Elemental Mercury Vapor Occurring after Reductive Loss from Foliage. *Environ. Sci. Technol.* **53**, 651–660 (2019).

20. Gratz, L. E., Keeler, G. J., Blum, J. D. & Sherman, L. S. Isotopic Composition and Fractionation of Mercury in Great Lakes Precipitation and Ambient Air. *Environ. Sci. Technol.* **44**, 7764–7770 (2010).
21. Demers, J. D., Sherman, L. S., Blum, J. D., Marsik, F. J. & Dvonch, J. T. Coupling atmospheric mercury isotope ratios and meteorology to identify sources of mercury impacting a coastal urban-industrial region near Pensacola, Florida, USA: Atmospheric mercury isotope ratios. *Glob. Biogeochem. Cycles* **29**, 1689–1705 (2015).
22. Fu, X., Marusczak, N., Wang, X., Gheusi, F. & Sonke, J. E. Isotopic composition of gaseous elemental mercury in the free troposphere of the Pic du Midi Observatory, France. *Environ. Sci. Technol.* **50**, 5641–5650 (2016).
23. Yu, B. *et al.* Isotopic Composition of Atmospheric Mercury in China: New Evidence for Sources and Transformation Processes in Air and in Vegetation. *Environ. Sci. Technol.* **50**, 9262–9269 (2016).
24. Yu, B. *et al.* New evidence for atmospheric mercury transformations in the marine boundary layer from stable mercury isotopes. *Atmospheric Chem. Phys.* **20**, 9713–9723 (2020).
25. Kurz, A. Y., Blum, J. D., Gratz, L. E. & Jaffe, D. A. Contrasting controls on the diel isotopic variation of Hg0 at two high elevation sites in the western United States. *Environ. Sci. Technol.* **54**, 10502–10513 (2020).
26. Sherman, L. S., Blum, J. D., Keeler, G. J., Demers, J. D. & Dvonch, J. T. Investigation of Local Mercury Deposition from a Coal-Fired Power Plant Using Mercury Isotopes. *Environ. Sci. Technol.* **46**, 382–390 (2012).
27. Chen, J., Hintelmann, H., Feng, X. & Dimock, B. Unusual fractionation of both odd and even mercury isotopes in precipitation from Peterborough, ON, Canada. *Geochim. Cosmochim. Acta* **90**, 33–46 (2012).
28. Wang, Z. *et al.* Mass-dependent and mass-independent fractionation of mercury isotopes in precipitation from Guiyang, SW China. *Comptes Rendus Geosci.* **347**, 358–367 (2015).
29. Fu, X. *et al.* Domestic and Transboundary Sources of Atmospheric Particulate Bound Mercury in Remote Areas of China: Evidence from Mercury Isotopes. *Environ. Sci. Technol.* **53**, 1947–1957 (2019).
30. Jiskra, M. *et al.* Mercury stable isotopes constrain atmospheric sources to the ocean. *Nature* **597**, 678–682 (2021).
31. Gehrke, G. E., Blum, J. D. & Meyers, P. A. The geochemical behavior and isotopic composition of Hg in a mid-Pleistocene western Mediterranean sapropel. *Geochim. Cosmochim. Acta* **73**, 1651–1665 (2009).
32. Brown, G., Sleeper, K., Johnson, M. W., Blum, J. D. & Cizdziel, J. V. Mercury concentrations, speciation, and isotopic composition in sediment from a cold seep in the northern Gulf of Mexico. *Mar. Pollut. Bull.* **77**, 308–314 (2013).
33. Ogrinc, N., Hintelmann, H., Kotnik, J., Horvat, M. & Pirrone, N. Sources of mercury in deep-sea sediments of the Mediterranean Sea as revealed by mercury stable isotopes. *Sci. Rep.* **9**, 11626 (2019).
34. Zambardi, T., Sonke, J. E., Toutain, J. P., Sortino, F. & Shinohara, H. Mercury emissions and stable isotopic compositions at Vulcano Island (Italy). *Earth Planet. Sci. Lett.* **277**, 236–243 (2009).
35. Sun, R. *et al.* Historical (1850–2010) mercury stable isotope inventory from anthropogenic

sources to the atmosphere. *Elem. Sci. Anthr.* **4**, 000091 (2016).

36. Si, M. *et al.* Measurement of atmospheric mercury over volcanic and fumarolic regions on the North Island of New Zealand using passive air samplers. *ACS Earth Space Chem.* **4**, 2435–2443 (2020).
37. Sun, R. *et al.* Modelling the mercury stable isotope distribution of Earth surface reservoirs: Implications for global Hg cycling. *Geochim. Cosmochim. Acta* **246**, 156–173 (2019).
38. Sun, R. *et al.* Mercury isotope evidence for marine photic zone euxinia across the end-Permian mass extinction. *Commun. Earth Environ.* **4**, 1–11 (2023).
39. Amos, H. M. *et al.* Observational and Modeling Constraints on Global Anthropogenic Enrichment of Mercury. *Environ. Sci. Technol.* **49**, 4036–4047 (2015).
40. Horowitz, H. M. *et al.* A new mechanism for atmospheric mercury redox chemistry: implications for the global mercury budget. *Atmospheric Chem. Phys.* **17**, 6353–6371 (2017).
41. Zhang, Y. *et al.* An updated global mercury budget from a coupled atmosphere-land-ocean model: 40% more re-emissions buffer the effect of primary emission reductions. *One Earth* **6**, 316–325 (2023).
42. Amos, H. M., Jacob, D. J., Streets, D. G. & Sunderland, E. M. Legacy impacts of all-time anthropogenic emissions on the global mercury cycle. *Glob. Biogeochem. Cycles* **27**, 410–421 (2013).
43. AMAP/UN Environment. *Technical Background Report for the Global Mercury Assessment 2018*. viii + 426 pp (2019).
44. Moynier, F. *et al.* The Mercury Isotopic Composition of Earth's Mantle and the Use of Mass Independently Fractionated Hg to Test for Recycled Crust. *Geophys. Res. Lett.* **48**, (2021).
45. Dal Corso, J. *et al.* Permo–Triassic boundary carbon and mercury cycling linked to terrestrial ecosystem collapse. *Nat. Commun.* **11**, 2962 (2020).
46. Zhao, H. *et al.* Mercury isotope evidence for regional volcanism during the Frasnian-Famennian transition. *Earth Planet. Sci. Lett.* **581**, 117412 (2022).
47. Nolan, M., Xiao, S., Gill, B. C., Jones, D. & Zhou, C. Evaluación de la Formación ediacárica de Doushantuo: mejora de la correlación estratigráfica de las pizarras negras de Doushantuo superior a partir del contenido en mercurio. *Estud. Geológicos* **75**, 107 (2019).
48. Fan, H. *et al.* Mercury isotopes track the cause of carbon perturbations in the Ediacaran ocean. *Geology* **49**, 248–252 (2021).
49. Jiang, G., Shi, X., Zhang, S., Wang, Y. & Xiao, S. Stratigraphy and paleogeography of the Ediacaran Doushantuo Formation (ca. 635–551Ma) in South China. *Gondwana Res.* **19**, 831–849 (2011).
50. Sanei, H., Grasby, S. E. & Beauchamp, B. Latest Permian mercury anomalies. *Geology* **40**, 63–66 (2012).
51. Percival, L. M. E. *et al.* Mercury evidence for pulsed volcanism during the end-Triassic mass extinction. *Proc. Natl. Acad. Sci.* **114**, 7929–7934 (2017).
52. Grasby, S. E., Them, T. R., Chen, Z., Yin, R. & Ardakani, O. H. Mercury as a proxy for volcanic emissions in the geologic record. *Earth-Sci. Rev.* **196**, 102880 (2019).
53. Shen, J. *et al.* Evidence for a prolonged Permian–Triassic extinction interval from global marine mercury records. *Nat. Commun.* **10**, 1563 (2019).
54. Pyle, D. M. & Mather, T. A. The importance of volcanic emissions for the global atmospheric mercury cycle. *Atmos. Environ.* **37**, 5115–5124 (2003).

55. Condon, D. *et al.* U-Pb Ages from the Neoproterozoic Doushantuo Formation, China. *Science* **308**, 95–98 (2005).
56. Wang, X. *et al.* Mercury anomalies across the Cryogenian-Ediacaran boundary in South China. *Precambrian Res.* **379**, 106771 (2022).
57. Wu, Y. *et al.* Global Hg cycle over Ediacaran–Cambrian transition and its implications for environmental and biological evolution. *Earth Planet. Sci. Lett.* **587**, 117551 (2022).
58. Sherman, L. S. *et al.* Mercury isotopic composition of hydrothermal systems in the Yellowstone Plateau volcanic field and Guaymas Basin sea-floor rift. *Earth Planet. Sci. Lett.* **279**, 86–96 (2009).
59. Chen, J., Hintelmann, H. & Dimock, B. Chromatographic pre-concentration of Hg from dilute aqueous solutions for isotopic measurement by MC-ICP-MS. *J. Anal. At. Spectrom.* **25**, 1402 (2010).
60. Zheng, W. *et al.* Mercury stable isotope fractionation during abiotic dark oxidation in the presence of thiols and natural organic matter. *Environ. Sci. Technol.* **53**, 1853–1862 (2019).
61. Blum, J. D., Sherman, L. S. & Johnson, M. W. Mercury isotopes in Earth and environmental sciences. *Annu. Rev. Earth Planet. Sci.* **42**, 249–269 (2014).
